# Efficacy of a Web-Based Intervention for Depressive Disorders: Three-Arm Randomized Controlled Trial Comparing Guided and Unguided Self-Help With Waitlist Control

**DOI:** 10.2196/34330

**Published:** 2022-04-04

**Authors:** Rico Krämer, Lea Köhne-Volland, Anna Schumacher, Stephan Köhler

**Affiliations:** 1 Department of Psychiatry and Neuroscience Charité – Universitätsmedizin Berlin Berlin Germany; 2 Department of Psychology Technische Universität Chemnitz Germany; 3 Department of Psychology Sigmund-Freud Privat Universität Berlin Germany

**Keywords:** major depressive disorder, online intervention, blended treatment, public health, routine practice, randomized controlled trial, depression, disorder, intervention, treatment, efficacy, self-help, guidance

## Abstract

**Background:**

Digital health apps are efficacious treatment options for mild-to-moderate depressive disorders. However, the extent to which psychological guidance increases the efficacy of these apps is controversial.

**Objective:**

We evaluated the efficacy of a web-based intervention, called Selfapy, for unipolar depression. We also investigated differences between psychotherapist-guided and unguided versions.

**Methods:**

Selfapy is a cognitive behavioral therapy–based intervention for depressive disorders. Participants with mild-to-severe depressive disorders were assigned randomly to participate in either guided (weekly 25-minute duration telephone calls) intervention, unguided version, or waiting list (control group) for 12 weeks. We assessed depressive symptoms at the start of the study, midway through the intervention (6 weeks), at the end of the intervention (12 weeks), and at follow-up (6 months). The main outcome was difference in the Beck Depression Inventory score between the start of the study and the end of the intervention. Secondary outcomes were the Quick Inventory of Depressive Symptomatology—Self Report, the Hamilton Rating Depression Scale, and the Beck Anxiety Inventory.

**Results:**

Of 401 participants, 301 participants (75.1%) completed the intervention. Changes in the Beck Depression Inventory from baseline differed significantly between groups at the postintervention (*F*_2,398_=37.20, *P*<.001). The reductions in scores for both guided and unguided intervention groups were greater than that for the control group, with large between-group effect sizes (guided vs control: *d*=1.63, 95% CI 1.37 to 1.93; unguided vs control: *d*=1.47, 95% CI 1.22 to 1.73) at postintervention. No significant differences were found between guided and unguided intervention groups (*P*=.18). At follow-up (6 months), treatment effects on the primary outcome were maintained for both intervention groups (guided: *F*_1,194_=0.62, *P*>.999; unguided: *F*_1,176_=0.13, *P*>.999).

**Conclusions:**

Both guided and unguided versions of the intervention were highly effective in reducing depressive symptoms. Follow-up data suggest that these effects could be maintained. The guided version was not superior to the unguided version.

**Trial Registration:**

German Clinical Trials Register DRKS00017191; https://tinyurl.com/2p9h5hnx

**International Registered Report Identifier (IRRID):**

RR2-10.1186/s13063-021-05218-4

## Introduction

### Background

With more than 300 million people affected worldwide, unipolar depression is a common mental disorder [1]. Depressive disorders reduce quality of life for affected persons and are linked to an increased prevalence of suicide and a shortened lifespan [2,3]. However, access to treatment is limited, which represents an obstacle in the care of people with depressive disorders. Health care systems can only rarely give necessary acute help, such as immediate access to a psychotherapist [4]. In Germany, it takes approximately 20 weeks to obtain outpatient psychotherapeutic treatment [5].

In addition to evidenced-based treatments for depressive disorders, such as psychotherapy and pharmacotherapy, web-based interventions are becoming increasingly important in the treatment of depressive disorders. Web-based interventions based on cognitive behavioral therapy are suitable due to their structured and standardized approach, their focus on psychoeducation, and the homework tasks assigned in-between treatment sessions [6]. Various forms of web-based interventions exist, which differ in terms of the level of guidance that they provide to the participant. The guided forms of web-based interventions can involve support from a psychotherapist via email, chat, or telephone. Unguided forms of web-based interventions usually do not include personal contact.

The use of web-based interventions in the treatment of depressive disorders has been deemed efficacious in several controlled studies [7-9] and meta-analyses [10-12]. In one meta-analysis [10], self-guided web-based cognitive behavioral therapy was found to be more effective than the control treatment in reducing depressive symptoms severity (*β*=−0.21; Hedges *g=*0.27) and treatment response (*β*=0.53; odds ratio 1.95, 95% CI 1.52 to 2.50). In a recent systematic review and individual patient data network meta-analysis of 39 randomized control trials, Karyotaki et al [12] made the distinction between guided and unguided web-based cognitive behavioral therapy. Both guided (PHQ-9 score: mean difference −1.7, 95% CI −2.3 to −1.1) and unguided (PHQ-9 score: mean difference −0.9, 95% CI −1.5 to −0.3) were more efficacious in reducing depressive symptoms than treatment as usual, and both guided (PHQ-9 score: mean difference −3.3, 95% CI −3.9 to −2.6) and unguided (PHQ-9 score: mean difference −2.5, 95% CI −3.2 to −1.8) were more efficacious in reducing depressive symptoms than waitlist control [12]; guided web-based cognitive behavioral therapy was also more effective than unguided web-based cognitive behavioral therapy postintervention (PHQ-9 score: mean difference −0.8; 95% CI −1.4 to −0.2), however, not at follow-up at 6 or 12 months. Baseline severity of depressive symptoms was a modifying factor, with better effects for guided web-based cognitive behavioral therapy for patients with baseline PHQ-9 scores greater than 9 [12]. However, Karyotaki et al [12] used varying definition of guidance between the studies and only 6 trials included in the meta-analysis directly compared guided to unguided web-based cognitive behavioral therapy within a single trial.

### Objectives

We aimed to evaluate the efficacy of guided and unguided versions of a web-based intervention, called Selfapy, to investigate the effect of psychological guidance in web-based interventions. In a randomized controlled trial, participants were allocated to 3 treatment groups: guided, unguided, and control.

### Hypotheses

We hypothesized that participants in the 3-month Selfapy program would experience a greater reduction in depressive symptoms than the control group, and we hypothesized that participants in the guided version would experience a greater reduction in depressive symptoms than participants in the unguided version.

### Secondary Hypotheses

We hypothesized that a greater reduction in depressive symptoms and anxiety symptoms would be present in both intervention groups after the 3-month Selfapy program than that in the control group.

## Methods

### Recruitment

Participants with depressive symptoms were recruited via the Selfapy website, advertisements in social media and numerous information brochures from health insurance companies. The recruitment took place throughout all of Germany. The central recruiting tool was a study website through which interested individuals could register their participation. This preregistered trial was conducted according to the study protocol [13].

### Ethical Standards

The study was approved by the ethics committee of the medical faculty of the *Charité* University Medicine Berlin (EA/047/19). All procedures contributing to this work comply with the ethical standards of the relevant national and institutional committees on human experimentation and with the Helsinki Declaration of 1975, as revised in 2008 [14].

### Inclusion and Exclusion Criteria

Potential participants were screened by telephone. Eligibility for participation in our study was assessed by conducting a diagnostic interview using the Mini International Neuropsychiatric Interview (MINI [15]), the Hamilton Rating Depression Scale (HRSD-24) [16] (score ≥8), and by collecting personal data. All MINI and HRSD-24 interviews were conducted by trained interviewers (psychologists and medical students, trained at the *Charité* Department of Psychiatry and Psychotherapy). The inclusion criteria were (1) age 18 to 65 years; (2) sufficient German-language skills to use and understand the web-based intervention (determined by interviewers); (3) reliable internet access; (4) a Beck Depression Inventory (BDI-II) [17] score ≥13; (5) willingness to provide electronic data; and (6) diagnosis of a major depressive disorder or dysthymia based on the MINI, in accordance with the International Statistical Classification of Diseases tenth revision (ICD-10: F32, F33, F34).

Exclusion criteria were (1) diagnoses of a bipolar disorder or schizophrenia; (2) acute psychotic symptoms; (3) current substance dependence (within the past 6 months) or withdrawal syndrome (ICD-10: F1x2, F1x3); (4) acute suicidality (assessed using HRSD-24; individuals were excluded if they had a score ≥3 on suicidality items). Individuals who were excluded from the study due to illness severity were advised to seek professional help. Additional details have been previously published [13].

### Randomization and Blinding

**Figure 1:**
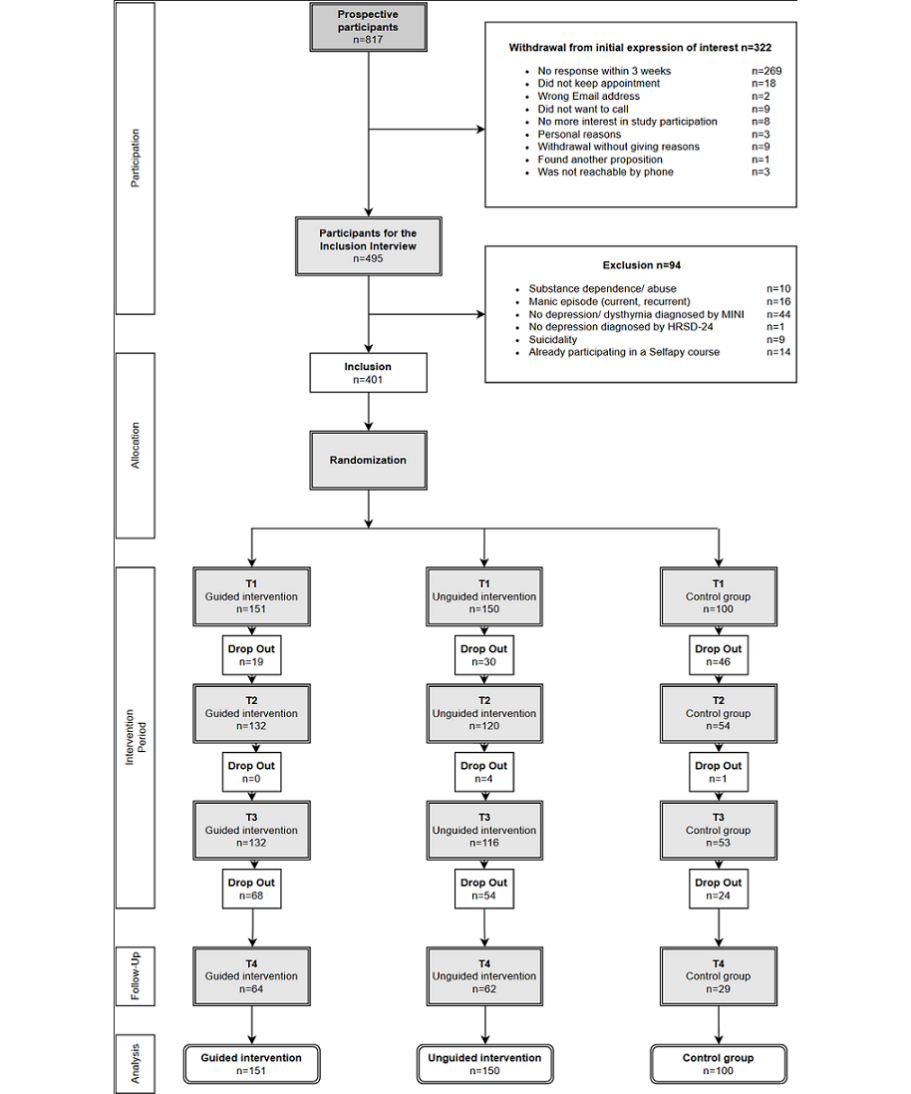
Participant flowchart.

Participants meeting eligibility criteria were randomly allocated to 3 groups (Figure 1). Participants were allocated in a 3:3:2 ratio (guided group: n=151, unguided group: n=150, control group: n=100). Block randomization was performed by an independent researcher using a random number assignment plan with a computer-controlled random number generator (Randlist, version 1.2).

Participants either received immediate access to the guided version of the program, immediate access to the unguided version of the program, or delayed access (24 weeks) to their choice of the guided or unguided program (ie, control group). Participants were informed via email about the result of the allocation process. Individuals in the intervention groups received an email with a link and their unique access code to register and start the intervention immediately. Individuals in the control group also received an email with a link to the assessment material. Therefore, participants enrolled themselves in the study. Diagnostic interviewers were blind to the assigned group of individuals.

### Intervention

The web-based intervention aimed to treat depressive symptoms in individuals with mild-to-moderate depressive disorders, with instructions on evidence-based methods and exercises in the areas of cognitive behavioral therapy, systemic therapy, and mindfulness training. The intervention consisted of 6 core modules and 6 additional optional in-depth modules representing different psychotherapeutic approaches (Multimedia Appendix 1), each of which could be completed in 10 to 60 minutes, depending on the user’s reading speed, interest, motivation, and individual path through the program. The modules could be accessed repeatedly during the intervention period. The course was designed to engage the user in active exercises, provide helpful and interesting content, and encourage self-reflection. In addition, the intervention included short questionnaires to assess current mood, which allowed the mood trajectory to be visualized over the course of therapy. Furthermore, individual goals could be set and reviewed. The program provided the user with printable summaries and worksheets for each exercise. Optionally, the user could receive reminder emails to use the course and reiterate program content.

Participants in both intervention groups used the same web-based course for 12 weeks, and access to course content was also available after the 12-week intervention period until follow-up. Telephone or chat support was only offered during the treatment period. Participants in the intervention and control groups were not influenced or advised to change their existing treatment patterns and were free to seek pharmacological or psychological treatments to meet the reality of care.

### Guided Group

In the guided version of Selfapy, the participants received personal guidance by a psychotherapist-in-training (17 behavior therapists and depth psychologists in training, registered at German institutes) for the entire duration of the program. The topics discussed were in line with the course content (Multimedia Appendix 1). The therapists were able to set their own focal points within the weekly topic and the associated exercises. At the start of the program, the psychotherapist-in-training and the participant got to know each other, and psychotherapist-in-training and the participant had weekly telephone calls (25 to 30 minutes duration) throughout the intervention period. The telephone calls focused on discussing and reflecting on the exercises of each module. Each module addressed issues such as resources, behavioral activation, self-esteem, and automatic thoughts. All therapists who guided the participants received a 1-hour training session that included: general information on the study; risks and their mitigation; a discussion of the contingency plan; information on handling and documenting dropouts; and information on the standardization of telephone calls. The focus of the guided version was to support web-based intervention use.

### Unguided Group

In the unguided version of Selfapy, the intervention was independently followed. There was no option to have contact with a psychotherapist-in-training via telephone. However, a chat functionality allowed the participants to ask questions regarding the correct use of the course. Active asynchronous communication occurred only in the event of patient safety concerns. For an increase in acute symptoms or suicidality, a specific protocol [13], for all study groups, was followed to secure the safety of each participant.

### Control Group

During the 24-week waiting period, the control group received weekly standardized mindfulness exercises via email, with content comparable to that of a self-help mindfulness guide. A waitlist design with mood-stabilizing activities was chosen for the control group to control for changes related to treatment expectancy and to better mitigate loss of motivation compared with an untreated or passive waitlist control group [18]. These exercises were only available for the control group so that there was no content-related overlap between the intervention groups and the control group. The control group was also free to access other pharmacological and psychological treatments. After the 24-week period, participants from the control group were given access to the web-based intervention and allowed to choose which type of program (guided or unguided) they wished to participate in.

### Measurements

Depressive symptoms were evaluated using the BDI-II (primary outcome), Quick Inventory of Depressive Symptomatology—Self Report (QIDS-SR-16) [19] and the observer-rated HRSD-24. The Beck Anxiety Inventory (BAI) [20] was used to measure changes in the self-assessment of anxiety symptoms (secondary outcome parameters). The primary and secondary outcome parameters were measured at the start of the intervention (T1), 6 weeks after the start of the intervention (T2), at the end of the intervention (12 weeks after the start of the intervention, T3), 24 weeks after the beginning of the intervention (follow-up, T4). All web-based questionnaires were completed independently by the participants.

### Statistical Analyses

Consistent with CONSORT (Consolidated Standards of Reporting Trials recommendations) [21], we conducted (1) intention-to-treat (which comprised observed and imputed data from all randomized participants, regardless of whether they used the intervention or activated their access vouchers to enter the program), and (2) per protocol (which comprised data from participants who completed pretreatment and postintervention assessments) analyses (Multimedia Appendix 2).

The primary endpoint was the decrease in depressive symptoms in the BDI-II between study entrance (T1) and the end of the intervention (T3). One-way analysis of variance (within-factor group) was performed to analyze differences in the decrease of depressive symptoms between the intervention groups.

Repeated measures analysis of variance was used to evaluate secondary endpoints and effects of group (guided vs unguided vs control) and time interaction. If significant effects were found, pairwise comparisons were carried out by applying Bonferroni correction (*P*<.016) for multiple testing. Results of the posthoc comparisons are presented as the mean with 95% CI and SD.

The Kolmogorov–Smirnov test was used to test for a normal distribution. Values for the mean and SD of each variable were calculated in addition to the Kolmogorov–Smirnov *Z*-value, and the asymptomatic significance (for both intervention groups) was specified. *P*<.05 indicated that the data did not have a normal distribution.

Independent 2-tailed *t* tests and chi-square tests were used to estimate the differences between groups in terms of demographics and sample characteristics at baseline. Values for the mean, 95% CI, and SD were calculated. Interim analyses were not undertaken. Due to the high dropout rate from T3 to T4, repeated measures analysis of variance was performed for the follow-up-analysis, including only those who completed.

Moderator analysis was used to analyze the influence of various sociodemographic variables on the primary outcome. Regression analysis was directed at explaining the changes in the BDI-II (the difference between T3 and T1 was used as a criterion). The predictors used were the BDI-II at baseline as well as potential moderators, assigned group, and sociodemographic variables (sex, age, relationship status, and number of children). All variables except age were dichotomous and coded as 0 or 1. The moderator variables were generated by multiplying the *z*-standardized BDI-II score at baseline with the dichotomous sociodemographic variables, the assigned group, and the *z*-standardized age. All dichotomous variables, assigned group, and *z*-standardized age were included as regressors. Subsequently, we used hierarchical linear regression, which had all predictors in the first block via the enter method and all moderators in the second block via the stepwise method.

Furthermore, response rates (decrease of BDI-II score from baseline of 50%), remission rates (postintervention BDI-II score ≤10 [22]), and the minimal clinical important difference (decrease of 17.5% of the BDI-II score from baseline [23]) for the primary outcome at postintervention were calculated and reported.

For the intention-to-treat analysis, missing values in the data were replaced using multiple imputation by chained equations (with *m*=5 imputations). The pooled data (the mean of all 5 imputations) were calculated using the data imputed by linear regression. Subsequently, scale values were determined from the imputed and existing values. After data imputation, imputed and observed results were compared. The pooled imputed values proved to be more conservative, therefore, the results of imputed data set were used to evaluate the outcome of the web-based intervention.

## Results

### General

Out of 401 participants, the number of dropouts at postintervention (T3, end of the intervention) was 100 (24.9%) for the BDI-II and the QIDS-SR-16, 128 (31.9%) for the HRSD-24, and 103 (25.7%) for the BAI, respectively.

### Characteristics

Upon study entrance, 353 out of 401 randomized participants (88.0%) fulfilled the diagnostic criteria for a current major depressive episode (MINI interview), and 53 (13.2%) for dysthymia (Multimedia Appendix 3). Data at baseline indicated an average mild-to-severe level of depression in all participants (BDI-II: mean 30.5, SD 9.5, range 13-56). A one-way analysis of variance with the factor group revealed no differences at baseline (*F*_2,398_=0.23, *P*=.80). The mean age of participants was 37.1 years (SD 11.0).

For factor relationships, fewer participants (33/151, 22.0%) reported themselves to be married or living with a partner in the unguided group than in the control group (52/100, 52.0%; *χ*^2^_1_=8.25, *P*=.01), whereas no difference was shown between the guided and control groups (*χ*^2^_1_=1.56, *P*=.21) or between the guided and unguided groups (*χ*^2^_1_=2.97, *P*=.08). More participants were employed in the guided group (82/151, 54.3%) and the unguided group (86/150, 57.3%) compared to those in the control group (57/100, 57.0%; guided vs control: *χ*^2^_1_=9.12, *P*=.01; unguided vs control: *χ*^2^_1_=18.98, *P*<.001), while there was no difference between the guided and unguided groups (*χ*^2^_1_=1.76, *P*=.18). More participants in the control group (25/100, 25.0%) were trainees than those in the guided group (12/151, 7.9%; *χ*^2^_1_=5.68, *P*=.01) or unguided group (6/150, 4.0%; *χ*^2^_1_=12.62, *P*<.001), while there was no difference between the guided and unguided groups (*χ*^2^_1_=1.27, *P*=.26). Lastly, more participants in the control group (14/100, 14.0%) than in the unguided group (3/150, 2.0%; *χ*^2^_1_=6.55, *P*=.05) reported other occupations.

Analyses of the other sociodemographic variables did not reveal a significant difference between groups (sex: *P*=.81, number of children: *P*=.93).

Being on waitlist (*χ*^2^_2_=6.76, *P*=.03), use of antidepressant medication both currently (*χ*^2^_2_=7.31, *P*=.03) and in the year before the intervention (*χ*^2^_2_=10.25, *P*=.006) differed at baseline, with more participants in the guided group having taken antidepressants than the unguided group in the year before the intervention (*χ*^2^_1_=9.36, *P*=.002) and currently (*χ*^2^_1_=6.56, *P*=.01). There were no differences compared to the control group in the year before the intervention (guided vs control: *χ*^2^_1_=0.66, *P*=.42; unguided vs control: *χ*^2^_1_=3.2, *P*=.07) and currently (guided vs control: *χ*^2^_1_=0.47, *P*=.49; unguided vs control: *χ*^2^_1_=2.11, *P*=.15). The groups did not differ with respect to current psychotherapy at baseline (*χ*^2^_2_=1.50, *P*=.47).

**Table 1:** Sociodemographic characteristics of the study cohort at baseline.

Characteristic		Guided (n=151)	Unguided (n=150)	Control (n=100)	Total sample (n=401)
**Sex, n (%)**					
	Female	126 (83.4)	126 (84.0)	81 (81.0)	333 (83.0)
	Male	25 (16.6)	24 (16.0)	19 (19.0)	68 (17.0)
Age (years), mean (SD)		38 (10.7)	37 (10.8)	36 (11.9)	37 (11.0)
**Relationship status, n (%)**					
	Married or living with a partner	54 (35.8)	33 (22.0)	52 (52.0)	139 (34.7)
	Not living with a partner	19 (12.6)	8 (5.3)	19 (19.0)	46 (11.5)
	Single	68 (45.0)	75 (50.0)	26 (26.0)	169 (42.1)
	Not reported	10 (6.6)	34 (22.7)	3 (3.0)	47 (11.7)
**Children, n (%)**					
	Yes	31 (20.5)	33 (22.0)	11 (11.0)	75 (18.7)
	No	89 (58.9)	99 (66.0)	37 (37.0)	225 (56.1)
	Not reported	31 (20.5)	18 (12.0)	52 (52.0)	101 (25.2)
**Professional qualification, n (%)**					
	Still in professional training	11 (7.3)	6 (4.0)	16 (16.0)	33 (8.2)
	Apprenticeship	28 (18.5)	19 (12.7)	25 (25.0)	72 (18.0)
	Master or vocational school	17 (11.3)	15 (10.0)	9 (9.0)	41 (10.2)
	University or university of applied sciences	39 (26.0)	45 (30.0)	30 (30.0)	114 (28.4)
	Without professional training	15 (9.9)	18 (78.7)	8 (8.0)	41 (10.2)
	Other professional training	2 (1.3)	0 (0.0)	8 (8.0)	10 (2.5)
	Not reported	39 (25.8)	47 (31.3)	4 (4.0)	90 (22.4)
**Occupation, n (%)**					
	Employee	82 (54.3)	86 (57.3)	57 (57.0)	225 (56.1)
	Self-employed	3 (2.0)	4 (2.7)	2 (2.0)	9 (2.2)
	Trainee	12 (7.9)	6 (4.0)	25 (25.0)	43 (10.7)
	Other	7 (4.6)	3 (2.0)	14 (14.0)	24 (6.0)
	Not reported	47 (31.3)	51 (34.0)	2 (2.0)	100 (24.9)

### Usage Data

A total of 301 participants received the intervention after baseline assessment. A mean of 9.4 (SD 2.3) modules were completed by each participant during the intervention period, and 254 participants (84.4%) completed the main course (Multimedia Appendix 3).

### Primary Outcome

Descriptive statistics for the for each assessment point are shown in Table 2 for completer and intention-to-treat samples. Kolmogorov-Smirnov tests did not reveal any violation of the normal distribution for BDI-II scores. One-way analysis of variance revealed a significant interaction (factor group) in the intention-to-treat sample (*F*_2,398_=37.20, *P*<.001). Posthoc pairwise comparisons with Bonferroni correction at postintervention (T3) revealed a significant higher reduction in depressive symptoms (BDI-II) in the guided group vs the control group (*P*<.001) and the unguided group vs the control group (*P*<.001). There was no significant difference (*P*=.18) between guided and unguided groups (Multimedia Appendix 4).

Within-group effect sizes for BDI-II (Table 3) were large both for the guided (*d=*1.44, 95% CI 1.21 to 1.68) and unguided (*d=*1.38, 95% CI 1.15 to 1.65) groups, whereas the control group showed no effect (*d=*0.07, 95% CI −0.21 to 0.37). Postintervention between-group effect sizes between the guided and control groups (*d=*1.63, 95% CI 1.37 to 1.93) and between the unguided and control groups (*d=*1.47, 95% CI 1.22 to 1.73) were large, whereas effect sizes between the guided and unguided groups were negligible (*d=*0.20, 95% CI −0.04 to 0.45).

**Table 2:** Assessment scores.

Outcome and group			Per protocol									Intention to treat				
		T1^a^			T2^b^			T3^c^			T2				T3	
		n		Mean (SD)	n	Mean (SD)	n		Mean (SD)	n			Mean (SD)	n		Mean (SD)
**Beck Depression Inventory-II**																
	Guided (n=151)	151		30.09 (9.18)	132	19.59 (6.60)	132		14.87 (8.77)	151			20.71 (6.98)	151		16.61 (9.55)
	Unguided (n=150)	150		30.54 (8.53)	120	20.44 (7.22)	116		15.86 (8.03)	150			22.51 (7.83)	150		18.49 (8.88)
	Control (n=100)	100		30.88 (10.74)	54	27.30 (7.05)	53		31.11 (8.30)	100			29.09 (6.39)	100		30.26 (6.97)
**Quick Inventory of Depressive Symptomatology – Self Report**																
	Guided (n=151)	151		17.41 (6.17)	132	11.14 (4.63)	132		6.53 (3.55)	151			11.62 (4.54)	151		7.33 (4.01)
	Unguided (n=150)	150		19.36 (5.44)	120	11.00 (3.71)	116		6.84 (4.09)	150			12.09 (4.03)	150		7.99 (4.31)
	Control (n=100)	100		18.55 (6.04)	54	17.17 (4.50)	53		20.15 (3.78)	100			16.90 (3.60)	100		17.88 (4.06)
**Hamilton Rating Depression Scale**																
	Guided (n=151)	151		23.23 (6.28)	N/A^d^	N/A	123		11.46 (6.81)	N/A			N/A	151		11.95 (6.50)
	Unguided (n=150)	150		23.22 (6.75)	N/A	N/A	70		12.19 (6.57)	N/A			N/A	150		14.75 (5.88)
	Control (n=100)	100		22.64 (6.76)	N/A	N/A	80		20.91 (8.78)	N/A			N/A	100		21.13 (8.20)
**Beck** **Anxiety Inventory**																
	Guided (n=151)	150		32.46 (11.32)	132	23.36 (11.97)	128		14.45 (9.06)	151			24.65 (11.79)	151		17.25 (11.05)
	Unguided (n=150)	150		34.09 (11.68)	120	20.43 (9.25)	118		16.22 (9.57)	150			23.27 (10.23)	150		19.98 (11.67)
	Control (n=100)	100		31.83 (14.14)	52	37.92 (9.62)	52		31.02 (7.51)	100			37.56 (7.98)	100		34.91 (7.56)

^a^T1 represents the start of the study.

^b^T2 represents the midpoint of the intervention (6 weeks after the start of the study)

^c^T3 represents the end of the intervention (12 weeks after the start of the study).

^d^N/A: not applicable. No data were available because the Hamilton Rating Depression Scale was not used at the midpoint assessment.

**Table 3:** Within- and between-group effect sizes for all groups in the intention-to-treat sample.

Measure and group			Within group, Cohen *d* (95% CI)				Between group (vs unguided), Cohen *d* (95% CI)					Between group (vs control), Cohen *d* (95% CI)		
			T1^a^–T2^b^		T1–T3^c^		T2		T3		T2			T3
**Beck Depression Inventory II**														
	Guided	1.15 (0.91, 1.40)		1.44 (1.21, 1.68)		0.24 (0.02, 0.48)		0.20 (−0.04, 0.45)		1.25 (0.99, 1.54)			1.63 (1.37, 1.93)	
	Unguided	0.98 (0.75, 1.25)		1.38 (1.15 1.65)		—^d^		—		0.92 (0.65, 1.20)			1.47 (1.22, 1.73)	
	Control	0.20 (−0.09, 0.47)		0.07 (−0.21, 0.37)		—		—		—			—	
**Quick Inventory of Depressive Symptomatology – Self Report**														
	Guided	1.07 (0.84, 1.33)		1.94 (1.68, 2.24)		0.11 (−0.10, 0.33)		0.16 (−0.06, 0.39)		1.29 (1.02, 1.53)			2.61 (2.28, 3.02)	
	Unguided	1.52 (1.28, 1.80)		2.32 (1.95, 2.72)		—		—		1.26 (1.04, 1.53)			2.36 (1.98, 2.82)	
	Control	0.33 (0.05, 0.63)		0.13 (−0.14, 0.45)		—		—		—			—	
**Hamilton Rating Depression Scale**														
	Guided	N/A^e^		1.76 (1.50, 2.05)		N/A		0.45 (0.21, 0.70)		N/A			1.24 (0.93, 1.59)	
	Unguided	N/A		1.34 (1.10, 1.61)		—		—		N/A			0.89 (0.62, 1.21)	
	Control	N/A		0.20 (−0.08, 0.48)		—		—		—			—	
**Beck** **Anxiety Inventory**														
	Guided	0.67 (0.44, 0.93)		1.35 (1.10, 1.64)		−0.13 (−0.37, 0.11)		0.241 (0.01, 0.46)		1.28 (1.04, 1.55)			1.87 (1.61, 2.20)	
	Unguided	0.99 (0.73, 1.25)		1.21 (0.94, 1.51)		—		—		1.56 (1.28, 1.86)			1.52 (1.25, 1.84)	
	Control	−0.50 (−0.79, −0.22)		−0.27 (−0.55, 0.01)		—		—		—			—	

^a^T1 represents the start of the study.

^b^T2 represents the midpoint of the intervention (6 weeks after the start of the study)

^c^T3 represents the end of the intervention (12 weeks after the start of the study).

^d^No data.

^e^Not available because the Hamilton Rating Depression Scale was not used at the midpoint assessment.

### Response and Remission Rate

Response, defined as the percentage of participants that had a reduction of depressive symptoms by 50% or more at postintervention (T3), was reached by 34.9% of all participants (n=140/401). In the guided group, the response rate was 48.3% (73/151), 43.3% (65/150) in the unguided group, and 2.0% (2/100) in the control group. Remission, defined as a postintervention BDI-II score of 12 or less, occurred in 25.4% of all participants (102/401) of the intention-to-treat sample. In the guided group, 39.7% of participants (60/151) reached remission, with 28.0% (42/150) in the unguided group. No participants in the control group reached remission.

### Minimal Clinical Important Difference

Overall, 63.1% (253/401) of participants in the intention-to-treat sample had depressive symptom reductions greater than the minimal clinical important difference, with 74.2% (n=112/151) for the guided group, 70.7% (106/150) for the unguided group, and 35.0% (35/100) for the control group. In comparison, both the guided group (*χ*^2^_1_=36.44, *P*<.001) and the unguided group (*χ*^2^_1_=29.61, *P*<.001) had significantly more occurrences of symptom improvement than the control group, whereas no difference was found between the intervention groups (*χ*^2^_1_=0.30, *P*=.58).

### Moderator Analysis

The regression analysis was conducted using the intention-to-treat sample. The number of data sets that could be used for the calculation was reduced to 279, due to missing values in the sociodemographic variables. The regression appeared to be unproblematic (Durbin-Watson-statistic 1.762 and collinearities <2.0). As the nonstandardized residuals had a mean of 0, homoscedasticity of the regression was indicated. The Kolmogorov-Smirnov-test was asymptotically significant (*P*=.03). The skewed distribution lay within the 5% confidence interval; the kurtosis lay slightly above. Based on the histogram, the normal distribution of residuals is accepted. In the first block of the hierarchical multiple linear regression analysis, a significant model was found (explained variance *r*^2^=0.592; *F*_6,272_=65.9, *P*<.001). Notably, age influenced the treatment outcome significantly—the older the participants, the greater the improvement in the BDI-II score (*b*=0.103; *β*=0.087, *t*=2.25, *P*=.02). In addition, a higher BDI-II score at T1 was associated with greater reduction in BDI-II score at T3 (*b*=−0.98, *β*=−0.632, *t*=−15.93, *P*<.001). In addition, being assigned to the control group was associated with a lower reduction in the BDI-II score at T3 (*b*=11.6, *β*=0.34, *t*=8.52, *P*<.001). The other variables (relationship status: *P*=.96; sex: *P*=.29; number of children: *P*=.90) did not significantly predict the outcome variables. In the second block of the hierarchical regression analysis, the moderators that are the interaction terms of the variables from the first block at baseline were included using the stepwise method. None was found to be significant, therefore no moderation effect was indicated by the analysis.

### Secondary Outcomes

Descriptive statistics of secondary outcomes are displayed in Table 2, and Table 3 shows within- and between-group effect sizes for all secondary outcome measures for the intention-to-treat sample. No violation of the normal distribution was identified for any of the secondary outcomes.

**Figure 2:**
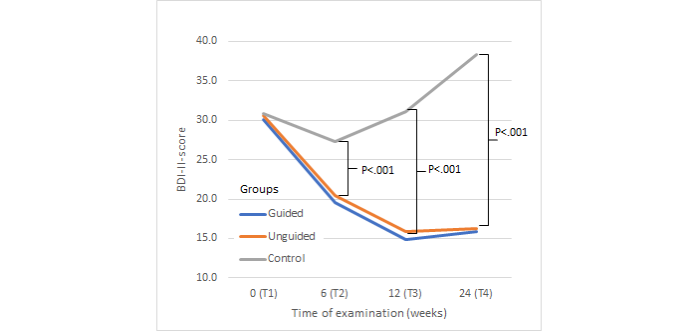
Change in depressive symptoms. BDI-II: Beck Depression Inventory-II.

Repeated measures analysis of variance revealed a significant main effect for the factor time—QIDS-SR-16 (*F*_3,1194_=200.08, *P*<.001, *η*^2^=0.25), HRSD-24 (*F*_2,796_=152.26, *P*<.001, *η*^2^=0.19), and BAI (*F*_2,796_=62.2 *P*<.001, *η*^2^=0.09). Additionally, we found a significant interaction (factors group × time) for all secondary measurements in the intention-to-treat sample—QIDS-SR-16 (*F*_6,1194_=33.2, *P*<.001, *η*^2^=0.10), HRSD-24 (*F*_4,796_=23.3, *P*<.001, *η*^2^=.07), and BAI (*F*_4,796_=30.4, *P*<.001, *η*^2^=0.09) (Figure 2).

**Figure 3:**
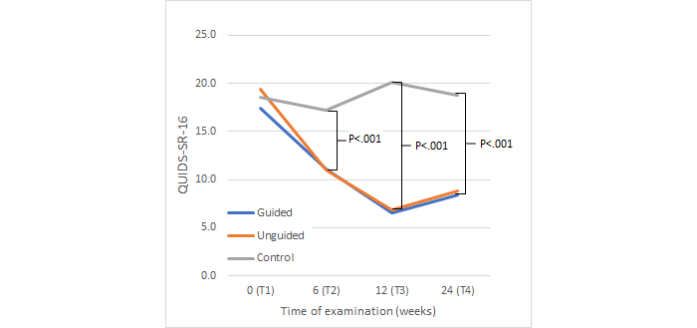
Change in depressive symptoms. QIDS-SR-16: Quick Inventory of Depressive Symptomatology—Self Report.

Bonferroni-adjusted posthoc analyses of the QIDS-SR-16 (Figure 3) revealed a greater reduction of depressive symptoms for both the guided group (*P*<.001) and the unguided group (*P*<.001) compared to the control group. However, no difference between the guided and unguided groups was found (*P*=.50).

**Figure 4:**
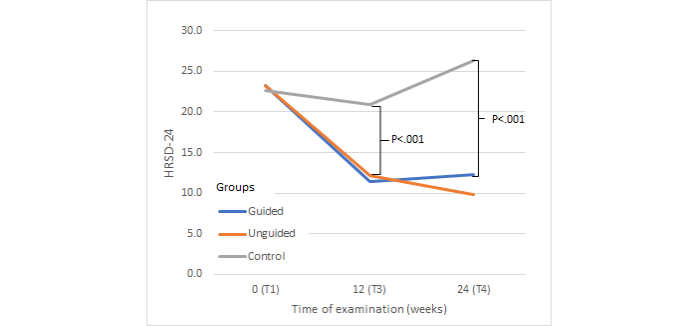
Change in depressive symptoms. HRSD-24: Hamilton Rating Depression Scale.

Similarly, posthoc analyses of the HRSD-24 (Figure 4) revealed a greater reduction of observer-rated depressive symptoms both for the guided group (*P*<.001) and the unguided group (*P*<.001) compared to the control group. A greater reduction in symptoms was found for the guided group compared to the unguided group (*P*=.001).

**Figure 5:**
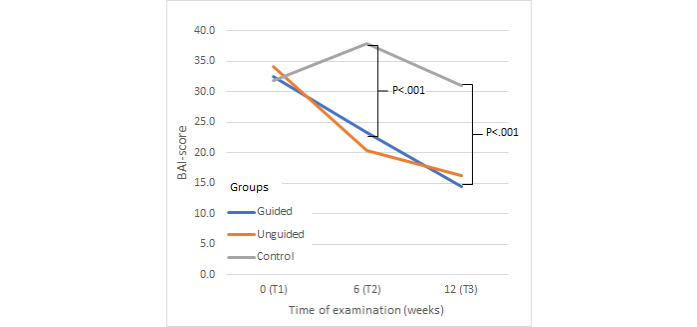
Change in anxiety symptoms. BAI: Beck Anxiety Inventory.

Finally, posthoc analyses of changes in BAI scores (Figure 5) revealed significantly greater reductions in anxiety symptoms in the guided (*P*<.001) and unguided groups (*P*<.001) compared to that of the control group. There was no significant difference between the guided group and unguided group (*P*=.08).

### Three-Month Follow-up

For the follow-up assessment 24 weeks after the start of the intervention (T4), repeated measures analysis of variances were carried out using per protocol data for the BDI-II, the QIDS-SR-16, and the HRSD-24. Data were available at follow-up for 155 (38.7%) for the BDI-II, 156 (38.9%) for the QIDS-SR-16, and 30 (7.5%) for the HRSD-24 out of all 401 participants (Table 4).

Repeated measures analysis of variance revealed a significant interaction (factors group × time) for the BDI-II (*F*_2,152_=3.7, *P*=.03, *η*^2^=0.02). Pairwise posthoc comparisons with Bonferroni-correction at follow-up (T4) showed a significant difference between the guided group (*P*<.001) and the unguided group (*P*<.001) compared to the control group, but not between guided and unguided (*P*>.999) demonstrating a symptom deterioration in the control group and maintenance of the treatment effects in both intervention groups. Compared to baseline (T1), the BDI-II scores at follow-up (T4) remained significantly lower for both the guided group (*d=*1.58) and the unguided group (*d=*1.88). Moreover, the remission rate at T4 in the BDI-II was 29.2% for the guided group and 21.3% for the unguided group. Repeated-measures analysis of variance did not reveal a significant interaction effect (group × time) for the QIDS-SR-16 (*F*_2,153_=3.32, *P*=.39, *η*^2^=0.02) or the HRSD-24 (*F*_2,19_=0.27, *P*=.77, *η*^2^=0.15).

**Table 4:** Change from T3 to follow-up (T4) with completer data for depression outcomes.

Outcome and group			T3^a^			T4^b^			Test statistics (T3-T4)				Within group
		n		Mean (SD)	n		Mean (SD)	*F* test (*df1,df2*)		*P* value	*η* ^2^	T1−T4, Cohen *d* (95% CI)	
**Beck Depression Inventory-II**													
	Guided	132		14.87 (8.77)	64		15.92 (8.79)	0.62 (1,194)		>.999	.00	1.58 (1.29, 1.95)	
	Unguided	116		15.86 (8.03)	62		16.29 (6.47)	0.13 (1,176)		>.999	.00	1.88 (1.59, 2.24)	
	Control	53		31.11 (8.30)	29		38.28 (9.88)	12.2 (1,80)		.002	.13	−0.72 (−1.35, −0.28)	
**Quick Inventory of Depressive Symptomatology – Self Report**													
	Guided	132		6.53 (3.55)	65		8.42 (4.80)	9.67 (1,195)		.006	.05	1.63 (1.31, 2.00)	
	Unguided	116		6.84 (4.09)	62		8.85 (5.23)	8.05 (1,176)		.02	.04	1.97 (1.60, 2.45)	
	Control	53		20.15 (3.78)	29		18.76 (5.14)	1.96 (1,80)		.50	.02	−0.04 (−0.43, 0.37)	
**Hamilton Rating Depression Scale**													
	Guided	123		11.46 (6.81)	17		12.24 (7.02)	0.19 (1,138)		>.999	.00	1.65 (0.94, 2.63)	
	Unguided	70		12.19 (6.57)	6		9.83 (5.19)	0.73 (1,74)		>.999	.01	2.22 (1.81, 3.03)	
	Control	80		20.91 (8.78)	7		26.29 (4.15)	2.55 (1,85)		.342	.03	−0.65 (−1.35, −0.09)	

^a^T3 represents the end of the intervention (12 weeks after the start of the study).

^b^T4 represents the follow-up point (24 weeks after the start of the study).

## Discussion

### Principal Results

We investigated the efficacy of a guided and unguided web-based intervention for the treatment of depressive disorders and found a significant improvement of depressive symptoms in the BDI-II (primary outcome) and the HRSD-24 for both intervention groups compared with those in the control group in the intention-to-treat sample, with large pre- and postintervention difference effect sizes observed for each intervention (BDI-II: guided group, *d=*1.44; unguided group, *d=*1.38; HRSD-24: guided group, *d=*1.76; unguided group, *d=*1.34). Similarly, self-reported measures for depression and anxiety symptoms revealed a significant pre- and postintervention difference intervention decrease in scores for both intervention groups (QIDS-SR-16: guided group, *d=*1.94 and unguided group, *d=*2.32; BAI: guided group, *d=*1.35 and unguided group, *d=*1.21) compared with those in the control group (QIDS-SR-16: *d=*0.13; BAI: *d=*−0.27).

In a similarly structured web-based intervention for depressive disorders, Meyer et al [24] investigated an unguided web-based cognitive behavioral therapy intervention for depressive disorders and found the web-based intervention to be highly efficacious (compared with waitlist control, pre- and postintervention differences using PHQ-9: *d=*1.32), which is comparable to our within-group effect sizes for the BDI-II.

In another trial with guided web-based intervention, Beiwinkel et al [25] investigated the efficacy of a 12-week web-based program for depression, with therapeutic support upon request (ie, psychologists gave feedback via telephone or email) compared with a waitlist control (which included unguided internet-based psychoeducation only) and reported pre-and postintervention BDI-II scores showed a significant reduction in depressive symptoms with large within-group effect size for the intervention group with guidance (*d=*1.42; control group: *d*=0.65).

In our randomized controlled trial, the treatment effects of both intervention groups were slightly higher than those in previous studies [24,25]. In [25], the intervention duration was also 12 weeks, but therapeutic support was offered only upon request. This approach might have stopped patients from seeking contact, and therefore, may have hampered the overall effect. Moreover, our interventions provided the option to contact a psychologist in both intervention groups (guided group: telephone calls; unguided group: standardized chat option) which, arguably, led to a better outcome. Other than differences in study design, the characteristics of the participants may also be a reason for the high effect sizes. Compared with other web-based cognitive behavioral therapy studies [24,26,27], the percentage of women in our randomized controlled trial (333/401, 83%) was higher (74.4% [24]). In general, women tend to seek web-based interventions more frequently than men [26,27]. Moreover, considerably more participants completed higher education, ie, university (28.4%) and vocational school (10.2%). A high education level is a predictor of high adherence to treatment [28] and a positive outcome of treatment [29] because participants are better able to transfer the content of a particular treatment to their life [30]. In individual patient data network meta-analysis, Furukawa et al [31] found a higher baseline severity of depressive symptoms associated with a better response to web-based interventions and being unemployed with a poorer outcome. Sex did not influence the response. We also found that baseline severity, treatment, and age (higher age with better outcome) were significant moderators of treatment outcome.

The efficacy of the web-based intervention over waitlist control is larger but consistent with previous literature on similar interventions for both guided (between-group effect size *d=*0.55 [24]) and unguided (between-group effect size *d=*0.57 [25]) web-based cognitive behavioral therapy. The response rate in our control group was relatively low compared to that in other web-based intervention trials (within-group effect size in BDI-II: *d*=0.07)—Meyer et al [24] found an average within-group effect size (*d=*0.71) in the primary outcome (PHQ-9) for the control group. Klein et al [32] reported for their control condition (care as usual alone) small effects in the pre- and postintervention comparison (PHQ-9: *d=*0.39). In contrast, Berger et al [33], also did not find significant changes between pre- and postintervention symptoms in their waiting list control group (BDI-II: *d=*0.14). Our results were similar. The low effects in our control group could be explained by many different reasons. Active waiting list condition, as in our control group, might have nocebo effects compared to no treatment condition [34]. Additionally, specific interventions in the waiting list condition may also have negative effects in internet interventions. For example, Furukawa et al [31] reported that relaxation training was even harmful compared to other components in web-based cognitive behavioral therapy.

The effects of could be maintained at 3-month follow-up. BDI-II scores remained significantly lower for the guided group (*d=*1.59) and unguided group (*d=*1.91) compared with baseline scores. Our findings are similar to those from previous research, which found web-based interventions have positive long-term effects [10].

We also investigated on the effects of guidance in web-based interventions. We found in both groups with different guidance as equally and highly effective to reduce depressive symptoms. Completely unsupported web-based interventions have been suggested to be less efficacious [8,12], associated with higher attrition rates [35], and to carry greater risks than supported interventions [34]. However, findings are to some extent heterogeneous: Berger et al [33] compared an unguided internet-based self-help program with the same intervention supported by a therapist and waitlist control group. Our comparison of guided and unguided did show differences, which, however, were not significant between the unguided group and guided group (mean groups difference at postintervention: *d*=0.24 in favor of guided self-help). In a recent investigation, Zagorscak et al [36] compared web-based cognitive behavioral therapy alone with therapy and semistandardized email feedback from psychologists. Again, between-group effects were nonsignificant across outcomes.

Regarding our study, the dose of psychological contact might not vary sufficiently to elicit substantial differences between the groups. Instead, both groups had contact with a therapist, although the unguided group could only reach out for non–content-related questions. Both groups also had a high main course completion rate, especially compared to nonguided web-based interventions in other studies [31]. Karyotaki et al [10] revealed that treatment adherence to web-based cognitive behavioral therapy (session completion rate) influenced the outcome. In contrast to the meta-analysis findings [12], we also did not find severity of depression to be a predictor for better outcome in the guided group.

### Strength and Limitations

Our study has several strengths. We included self- and observer ratings and included a follow-up assessment. Furthermore, we compared different forms of guidance. We also considered multiple aspects in our evaluation, such as completion rate and sociodemographic factors. However, there are also some limitations. First, using wide inclusion criteria, we acquired a heterogeneous study sample [37]. Second, the option to receive additional treatment impeded the attribution of treatment effects solely on the web-based intervention. Additional treatment (12 people were in therapy and 70 were receiving psychiatric treatment in both intervention groups) could have contributed to the effects and possibly caused a reduction in internal validity. Third, although conversations between psychotherapists and participants were standardized in the guided group, we had no insights into the actual conversations and whether the structure of the predetermined content was followed. Last, our sample size might have been too small to detect differences between the guided and unguided groups.

### Conclusions

The web-based intervention offers a highly efficacious and clinically relevant intervention for people with depressive disorders. Contrary to our hypothesis, the efficacy of the guided g and unguided intervention did differ. Our findings demonstrate the value and applicability of the Selfapy web-based intervention as a clinically significant treatment option for depressive disorders.

## Appendix

Multimedia Appendix 1 Course content.

Multimedia Appendix 2 Diagnoses according to Mini International Neuropsychiatric Interview (MINI).

Multimedia Appendix 3 Intervention usage data for the guided and unguided group.

Multimedia Appendix 4 Completer analysis.

Multimedia Appendix 5 CONSORT-EHEALTH checklist (V 1.6.2).

## Abbreviations

BAI: Beck Anxiety Inventory
BDI-II: Beck Depression Inventory-II
CONSORT: Consolidated Standards of Reporting Trials
HRSD-24: Hamilton Rating Depression Scale
ICD-10: International Statistical Classification of Diseases, tenth revision
MINI: Mini International Neuropsychiatric Interview
PHQ-9: Patient Health Questionnaire
QIDS-SR-16: Quick Inventory of Depressive Symptomatology—Self Report
