# Supplementary material for: Efficacy of a Web-Based Intervention for Depressive Disorders: Three-Arm Randomized Controlled Trial Comparing Guided and Unguided Self-Help With Waitlist Control
Source: JMIR Form Res. 2022 Apr 4;6(4):e34330. doi: 10.2196/34330 (PMC9016501; doi:10.2196/34330)
Supplement: Multimedia Appendix 1 [file formative_v6i4e34330_app1.docx]

| **Table 1.** Overview of course content **Module** | **Content** | **Exercises** |
| --- | --- | --- |
| Your beginning | A questionnaire is used to collect general data about this person and his/her problems. | Lifeline; problem cakes; miracle question |
| **Target selection** | | |
| First insights | In this module, the user is brought closer to the connection between thinking, feeling and acting. Based on this triangular connection, our course concept is explained to him/her. | Your personal triangles |
| Resources | Explanation and gathering of power sources. Prepare the behavioural activation. Different methods to assist users in collecting power sources. | Evaluation daily protocol; power source images; treasure chest; resource walk |
| Behavioural activation | Actively plan power sources during the day. | Activity plan |
| Automatic thoughts | These automatic thoughts and thinking patterns are what we want to address this week. | Automatic thoughts; core beliefs |
| Negative thoughts | Thinking about reality, thinking realistically | Think realistically |
| Self-esteem | Also refer to the resources. | - |
| Self-efficacy | Therefore, this week will make you aware of your recent successes. This will enable you to better allocate your power reserves and increase confidence in your abilities. | "Your personal achievements - be proud of yourself" |
| Social environment + stigma + social support | "This week's focus is on your environment and your social environment, and when we talk about sharing with our environment, another factor is very important: our social support can help us deal appropriately and successfully with our stress." | "Difficult communication -your backing" |
| Mindfulness + excursus: pleasure training | "Mindfulness is the focus of this week, and we want to work with you to cultivate a mindful approach to you and your environment, because a mindful attitude to life will help you to improve your stress management. Therefore, a well-being workout can help you to feel more joie de vivre and increase your well-being. | Mental note; mindfulness in everyday life; sitting meditation; body scan; your enjoyment moments |
| Solve problem | This week, we would like to introduce you to a training that will enable you to perceive a specific problem, capture potential responses in a problematic situation, and develop the competence to implement a particular course of action to solve this problem optimally. | Problem-solving training |
| Relapse cases | Finally, we want to tie up your anti-anxiety package with you. It should help you to recognize when the stress threatens to become too much again. We want to show you once more how you can best handle the stress when it cannot be avoided. | Risk situations; early warning symptoms; first aid plan; relapse protocol |
