# Supplementary material for: Efficacy of a Web-Based Intervention for Depressive Disorders: Three-Arm Randomized Controlled Trial Comparing Guided and Unguided Self-Help With Waitlist Control
Source: JMIR Form Res. 2022 Apr 4;6(4):e34330. doi: 10.2196/34330 (PMC9016501; doi:10.2196/34330)
Supplement: Multimedia Appendix 2 [file formative_v6i4e34330_app2.pdf]

Appendix 2. Diagnoses according to Mini International Neuropsychiatric Interview (MINI).

Diagnosis	Guided group (*N* = 151)		Unguided group (*N* = 150)		Control group (*N* = 100)		Total (*N* = 401)	
	N	%	*N*	%	*N*	%	*N*	%
Major depressive episode, current	143	94.7	132	88.0	78	78.0	353	88.0
Major depressive episode, lifetime	94	62.3	103	68.7	63	63.0	260	64.8
Dysthymia	9	6.0	20	13.3	24	24.0	53	13.2
Suicidality	16	10.6	10	6.7	3	3.0	29	7.2
Mania/hypomania, current	0	0.0	0	0.0	0	0.0	0	0.0
Mania/hypomania, lifetime	0	0.0	0	0.0	0	0.0	0	0.0
Panic disorder, current	39	25.8	24	16.0	9	9.0	72	18.0
Panic disorder, lifetime	88	58.3	60	40.0	29	29.0	177	44.1
Panic disorder without agoraphobia	19	12.6	12	8.0	7	7.0	38	9.5
Panic disorder with agoraphobia	26	17.2	18	12.0	4	4.0	48	12.0
Social anxiety disorder	23	15.2	20	13.3	9	9.0	52	13.0
Obsessive–compulsive disorder	57	37.7	58	38.7	32	32.0	147	36.7
Post-traumatic stress disorder	9	6.0	7	4.7	8	8.0	24	6.0
Alcohol dependence	17	11.3	18	12.0	10	10.0	45	11.2
Alcohol abuse	0	0.0	0	0.0	0	0.0	0	0.0
Drug dependence	1	0.7	0	0.0	4	4.0	5	1.2
Drug abuse	0	0.0	0	0.0	0	0.0	0	0.0
Psychotic disorder, current	0	0.0	0	0.0	1	1.0	1	0.2
Psychotic disorder, lifetime	0	0.0	0	0.0	0	0.0	0	0.0
Anorexia nervosa	0	0.0	0	0.0	0	0.0	0	0.0
Bulimia nervosa	0	0.0	2	1.3	1	1.0	3	0.7
Binge eating disorder	10	6.6	14	9.3	9	9.0	33	8.2
Generalized anxiety disorder	4	2.6	0	0.0	1	1.0	5	1.2
