# Supplementary material for: Efficacy of a Web-Based Intervention for Depressive Disorders: Three-Arm Randomized Controlled Trial Comparing Guided and Unguided Self-Help With Waitlist Control
Source: JMIR Form Res. 2022 Apr 4;6(4):e34330. doi: 10.2196/34330 (PMC9016501; doi:10.2196/34330)
Supplement: Multimedia Appendix 3 [file formative_v6i4e34330_app3.pdf]

Appendix 3. Intervention usage data for the guided and unguided group.

**Usage parameter**	Guided (*N* = 151)		Unguided (*N* = 150)		Total sample (*N* = 301)	
	M	*SD*	*M*	*SD*	*M*	*SD*
module progress	9.4	2.3	9.3	2.4	9.4	2.3
Main course completion rate[a], *N*, %	130	86.1%	124	83.2%	254	84.4%

Notes. [a] *Course completion of 6 core modules completed.*
