# Supplementary material for: Efficacy of a Web-Based Intervention for Depressive Disorders: Three-Arm Randomized Controlled Trial Comparing Guided and Unguided Self-Help With Waitlist Control
Source: JMIR Form Res. 2022 Apr 4;6(4):e34330. doi: 10.2196/34330 (PMC9016501; doi:10.2196/34330)
Supplement: Multimedia Appendix 4 [file formative_v6i4e34330_app4.pdf]

Appendix 4. Completer Analysis.

Primary Outcome

With regard to the primary outcome measure (BDI-II, completer sample), repeated-measures ANOVA revealed a significant main effect of time ($F_{(3,453)}$=160.83, *P*<.001, *η2*=0.18). Moreover, a significant interaction effect of group × time ($F_{(6,453)}$=32.11, *P*<.001, *η2*=0.12) was found.

Post hoc follow-up analysis with the Bonferroni correction was conducted to establish significant differences between groups. Significant differences were found between the guided group and control group (*P*<.001) and the unguided group and control group (*P*<.001). No significant difference was found between the guided group and unguided group (*P*=1.0).

Effect sizes (Cohen's *d*) for the BDI-II completer outcome for all groups are reported in Table 3.

Response, defined as the percentage of participants that had a reduction of depressive symptoms (BDI-II) by 50% or more at posttreatment (T3) was reached by 139 (46.2%) of all 301 completers. 55.3% (*n*=73 out of 132) in the guided group, 55.2% (*n*=64 out of 116) in the unguided group, and 3.8% (*n*=2 out of 53) in the control group.

Remission, defined as a BDI-II score of 10 or less at posttreatment in the completer sample was reached by81 (26.9%) of all 301 completers. 35.6% (*n*=47 out of 132) in the guided group, 29.3% (*n*=34 out of 116) in the unguided group. No participant in the control group reached remission.

Secondary Outcomes

Repeated-measures ANOVAs showed a significant interaction effect (group × time) between pre-treatment and post-treatment for QIDS-SR-16 and BAI measurements in the completer sample (QIDS-SR-16, $F_{(6,456)}$=18.63, *P*<.001, *η2*=0.15; BAI, $F_{(4,564)}$=22.22, *P*<.001, *η2*=0.09).

Also, a significant main effect for the factor time was found (QIDS-SR-16, $F_{(3,456)}$=65.76, *P*<.001; *η2*=0.24; BAI, $F_{(2,564)}$ = 72.23, *P*<.001, *η2*=0.15).

The HRSD-24 ($F_{(4,38)}$=2.54, *P*<.056, *η2=0.16)* showed no significant interaction effect (group × time) between pre-treatment and post-treatment in the completer sample. Also, no significant main effect for the factor time was found (HRSD-24, $F_{(2,38)}$=2.12, *P*=.13, *η2=0.07).*

Moreover, Bonferroni *post hoc* pairwise comparisons were conducted to reveal significant group differences for each outcome parameter.

A Bonferroni *post hoc* analysis of the QIDS-SR-16 scores was conducted and identified significant differences between both intervention groups and the control group (guided group and control group:

(*P*<.001); unguided group and control group: *P*<.001). Moreover, no significant difference was found between the guided group and unguided group (*P*=1.0).

Also, explorative Bonferroni *post hoc* analysis of the HRSD-24 scores demonstrated a significant difference between both intervention groups and control group (guided group and control group: *P*<.001; unguided group and control group: *P*<.001), with no significant difference between the guided group and unguided group (*P*=1.0).

Also, Bonferroni *post hoc* analysis of the BAI scores demonstrated a significant difference between both intervention groups and control group (guided group and control group: *P*<.001; unguided group and control group: *P*<.001), with no significant difference between the guided group and unguided group (*P*=.37).

Effect sizes (Cohen's *d*) for all secondary measurements for completer outcome for all groups are reported in Table 3.
