# Supplementary material for: Efficacy of a Web-Based Intervention for Depressive Disorders: Three-Arm Randomized Controlled Trial Comparing Guided and Unguided Self-Help With Waitlist Control
Source: JMIR Form Res. 2022 Apr 4;6(4):e34330. doi: 10.2196/34330 (PMC9016501; doi:10.2196/34330)
Supplement: Multimedia Appendix 5 [file formative_v6i4e34330_app5.pdf]

CONSORT-EHEALTH Checklist V1.6.2 Report	Manuscript Number	34330
**(based on CONSORT-EHEALTH V1.6), available at [http://tinyurl.com/consort-ehealth-v1-6].**		
**Date completed**		
3/23/2022 9:18:19		
**by**		
Stephan Köhler		
Efficacy of a Web-Based Intervention for Depressive Disorders: Three-Arm Randomized Controlled Trial Comparing Guided and Unguided Self-Help With Waitlist Control		
TITLE		
**1a-i) Identify the mode of delivery in the title**		
We evaluated the efficacy of a web-based intervention, called Selfapy, for unipolar depression		
**1a-ii) Non-web-based components or important co-interventions in title**		
**1a-iii) Primary condition or target group in the title**		
Efficacy of a Web-Based Intervention for Depressive Disorders		
ABSTRACT		
**1b-i) Key features/functionalities/components of the intervention and comparator in the METHODS section of the ABSTRACT**		
See Multimedia Appendix 1		
**1b-ii) Level of human involvement in the METHODS section of the ABSTRACT**		
Of 401 participants, 301 participants (75.1%) completed the intervention		
**1b-iii) Open vs. closed, web-based (self-assessment) vs. face-to-face assessments in the METHODS section of the ABSTRACT**		
We evaluated the efficacy of a web-based intervention, called Selfapy, for unipolar depression		
**1b-iv) RESULTS section in abstract must contain use data**		
Of 401 participants, 301 participants (75.1%) completed the intervention. Changes in the Beck Depression Inventory from baseline differed significantly between groups at the postintervention ($F_{2,398}$=37.20, P<.001). The reductions in scores for both guided and unguided intervention groups were greater than that for the control group, with large between-group effect sizes (guided vs control: d=1.63, 95% CI 1.37 to 1.93; unguided vs control: d=1.47, 95% CI 1.22 to 1.73) at postintervention		
**1b-v) CONCLUSIONS/DISCUSSION in abstract for negative trials**		
Both guided and unguided versions of the intervention were highly effective in reducing depressive symptoms. Follow-up data suggest that these effects could be maintained. The guided version was not superior to the unguided version		
INTRODUCTION		
**2a-i) Problem and the type of system/solution**		
We aimed to evaluate the efficacy of guided and unguided versions of a web-based intervention, called Selfapy, to investigate the effect of psychological guidance in web-based interventions.		
**2a-ii) Scientific background, rationale: What is known about the (type of) system**		
The use of web-based interventions in the treatment of depressive disorders has been deemed efficacious in several controlled studies [7-9] and meta-analyses [10-12].		

**Does your paper address CONSORT subitem 2b?**		
We aimed to evaluate the efficacy of guided and unguided versions of a web-based intervention, called Selfapy, to investigate the effect of psychological guidance in web-based interventions. In a randomized controlled trial, participants were allocated to 3 treatment groups: guided, unguided, and control.		
METHODS		
**3a) CONSORT: Description of trial design (such as parallel, factorial) including allocation ratio**		
Participants meeting eligibility criteria were randomly allocated to 3 groups (Figure 1). Participants were allocated in a 3:3:2 ratio (guided group: n=151, unguided group: n=150, control group: n=100). Block randomization was performed by an independent researcher using a random number assignment plan with a computer-controlled random number generator (Randlist, version 1.2).		
**3b) CONSORT: Important changes to methods after trial commencement (such as eligibility criteria), with reasons**		
No changes were applied.		
**3b-i) Bug fixes, Downtimes, Content Changes**		
**4a) CONSORT: Eligibility criteria for participants**		
Potential participants were screened by telephone. Eligibility for participation in our study was assessed by conducting a diagnostic interview using the Mini International Neuropsychiatric Interview (MINI [15]), the Hamilton Rating Depression Scale (HRSD-24) [16] (score ≥8), and by collecting personal data. All MINI and HRSD-24 interviews were conducted by trained interviewers (psychologists and medical students, trained at the Charité Department of Psychiatry and Psychotherapy). The inclusion criteria were (1) age 18 to 65 years; (2) sufficient German-language skills to use andunderstand the web-based intervention (determined by interviewers); (3) reliable internet access; (4) a Beck Depression Inventory (BDI-II) [17] score ≥13; (5) willingness to provide electronic data; and (6) diagnosis of a major depressive disorder or dysthymia based on the MINI, in accordance with the International Statistical Classification of Diseases tenth revision (ICD-10: F32, F33, F34).<br>Exclusion criteria were (1) diagnoses of a bipolar disorder or schizophrenia; (2) acute psychotic symptoms; (3) current substance dependence (within the past 6 months) or withdrawal syndrome (ICD-10: F1x2, F1x3); (4) acute suicidality (assessed using HRSD-24; individuals were excluded if they had a score ≥3 on suicidality items). Individuals who were excluded from the study due to illness severity were advised to seek professional help. Additional details have been previously published [13].		
**4a-i) Computer / Internet literacy**		

**4a-ii) Open vs. closed, web-based vs. face-to-face assessments:**		
The web-based intervention aimed to treat depressive symptoms in individuals with mild-to-moderate depressive disorders, with instructions on evidence-based methods and exercises in the areas of cognitive behavioral therapy, systemic therapy, and mindfulness training. The intervention consisted of 6 core modules and 6 additional optional in-depth modules representing different psychotherapeutic approaches (Multimedia Appendix 1), each of which could be completed in 10 to 60 minutes, depending on the user's reading speed, interest, motivation, and individual path through the program. The modules could be accessed repeatedly during the intervention period. The course was designed to engage the user in active exercises, provide helpful and interesting content, and encourage self-reflection. In addition, the intervention included short questionnaires to assess current mood, which allowed the mood trajectory to be visualized over the course of therapy		
**4a-iii) Information giving during recruitment**		
**4b) CONSORT: Settings and locations where the data were collected**		
Data collection is presented in the trial paper		
**4b-i) Report if outcomes were (self-)assessed through online questionnaires**		
Depressive symptoms were evaluated using the BDI-II (primary outcome), Quick Inventory of Depressive Symptomatology—Self Report (QIDS-SR-16) [19] and the observer-rated HRSD-24. The Beck Anxiety Inventory (BAI) [20] was used to measure changes in the self-assessment of anxiety symptoms (secondary outcome parameters). The primary and secondary outcome parameters were measured at the start of the intervention (T1), 6 weeks after the start of the intervention (T2), at the end of the intervention (12 weeks after the start of the intervention, T3), 24 weeks after the beginning of the intervention (follow-up, T4). All web-based questionnaires were completed independently by the participants.		
**4b-ii) Report how institutional affiliations are displayed**		
**5) CONSORT: Describe the interventions for each group with sufficient details to allow replication, including how and when they were actually administered**		
**5-i) Mention names, credential, affiliations of the developers, sponsors, and owners**		
**5-ii) Describe the history/development process**		
**5-iii) Revisions and updating**		

**5-iv) Quality assurance methods**		
**5-v) Ensure replicability by publishing the source code, and/or providing screenshots/screen-capture video, and/or providing flowcharts of the algorithms used**		
**5-vi) Digital preservation**		
The website of the intervention is www.selfapy.org		
**5-vii) Access**		
Participants in both intervention groups used the same web-based course for 12 weeks, and access to course content was also available after the 12-week intervention period until follow-up.		
**5-viii) Mode of delivery, features/functionalities/components of the intervention and comparator, and the theoretical framework**		
The content is displayed in Mult. App 2		
**5-ix) Describe use parameters**		
**5-x) Clarify the level of human involvement**		
**5-xi) Report any prompts/reminders used**		
Participants in both intervention groups used the same web-based course for 12 weeks, and access to course content was also available after the 12-week intervention period until follow-up. Telephone or chat support was only offered during the treatment period. Participants in the intervention and control groups were not influenced or advised to change their existing treatment patterns and were free to seek pharmacological or psychological treatments to meet the reality of care		
**5-xii) Describe any co-interventions (incl. training/support)**		
Potential participants were screened by telephone. Eligibility for participation in our study was assessed by conducting a diagnostic interview using the Mini International Neuropsychiatric Interview (MINI [15]), the Hamilton Rating Depression Scale (HRSD-24) [16] (score ≥8), and by collecting personal data. All MINI and HRSD-24 interviews were conducted by trained interviewers (psychologists and medical students, trained at the Charité Department of Psychiatry and Psychotherapy).		
**6a) CONSORT: Completely defined pre-specified primary and secondary outcome measures, including how and when they were assessed**		
The primary endpoint was the decrease in depressive symptoms in the BDI-II between study entrance (T1) and the end of the intervention (T3). One-way analysis of variance (within-factor group) was performed to analyze differences in the decrease of depressive symptoms between the intervention groups.		

**6a-i) Online questionnaires: describe if they were validated for online use and apply CHERRIES items to describe how the questionnaires were designed/deployed**		
**6a-ii) Describe whether and how “use” (including intensity of use/dosage) was defined/measured/monitored**		
A total of 301 participants received the intervention after baseline assessment. A mean of 9.4 (SD 2.3) modules were completed by each participant during the intervention period, and 254 participants (84.4%) completed the main course (Multimedia Appendix 3).		
**6a-iii) Describe whether, how, and when qualitative feedback from participants was obtained**		
**6b) CONSORT: Any changes to trial outcomes after the trial commenced, with reasons**		
Data collection is presented in the trial paper		
**7a) CONSORT: How sample size was determined**		
**7a-i) Describe whether and how expected attrition was taken into account when calculating the sample size**		
**7b) CONSORT: When applicable, explanation of any interim analyses and stopping guidelines**		
The primary endpoint was the decrease in depressive symptoms in the BDI-II between study entrance (T1) and the end of the intervention (T3). One-way analysis of variance (within-factor group) was performed to analyze differences in the decrease of depressive symptoms between the intervention groups.		
**8a) CONSORT: Method used to generate the random allocation sequence**		
Participants were allocated in a 3:3:2 ratio (guided group: n=151, unguided group: n=150, control group		
**8b) CONSORT: Type of randomisation; details of any restriction (such as blocking and block size)**		
Block randomization was performed by an independent researcher using a random number assignment plan with a computer-controlled random number generator (Randlist, version 1.2).		
**9) CONSORT: Mechanism used to implement the random allocation sequence (such as sequentially numbered containers), describing any steps taken to conceal the sequence until interventions were assigned**		
Block randomization was performed by an independent researcher using a random number assignment plan with a computer-controlled random number generator (Randlist, version 1.2).		
**10) CONSORT: Who generated the random allocation sequence, who enrolled participants, and who assigned participants to interventions**		

Potential participants were screened by telephone. Eligibility for participation in our study was assessed by conducting a diagnostic interview using the Mini International Neuropsychiatric Interview (MINI [15]), the Hamilton Rating Depression Scale (HRSD-24) [16] (score ≥8), and by collecting personal data. All MINI and HRSD-24 interviews were conducted by trained interviewers (psychologists and medical students, trained at the Charité Department of Psychiatry and Psychotherapy).		
**11a) CONSORT: Blinding - If done, who was blinded after assignment to interventions (for example, participants, care providers, those assessing outcomes) and how**		
**11a-i) Specify who was blinded, and who wasn’t**		
Diagnostic interviewers were blind to the assigned group of individuals.		
**11a-ii) Discuss e.g., whether participants knew which intervention was the “intervention of interest” and which one was the “comparator”**		
Participants in both intervention groups used the same web-based course for 12 weeks, and access to course content was also available after the 12-week intervention period until follow-up. Telephone or chat support was only offered during the treatment period. Participants in the intervention and control groups were not influenced or advised to change their existing treatment patterns and were free to seek pharmacological or psychological treatments to meet the reality of care.		
**11b) CONSORT: If relevant, description of the similarity of interventions**		
This question does not fit to our trial		
**12a) CONSORT: Statistical methods used to compare groups for primary and secondary outcomes**		
The primary endpoint was the decrease in depressive symptoms in the BDI-II between study entrance (T1) and the end of the intervention (T3). One-way analysis of variance (within-factor group) was performed to analyze differences in the decrease of depressive symptoms between the intervention groups. Repeated measures analysis of variance was used to evaluate secondary endpoints and effects of group (guided vs unguided vs control) and time interaction. If significant effects were found, pairwise comparisons were carried out by applying Bonferroni correction ($P<.016$) for multiple testing. Results of the posthoc comparisons are presented as the mean with 95% CI and SD. The Kolmogorov–Smirnov test was used to test for a normal distribution. Values for the mean and SD of each variable were calculated in addition to the Kolmogorov–Smirnov Z-value, and the asymptomatic significance (for both intervention groups) was specified. $P<.05$ indicated that the data did not have a normal distribution.		
**12a-i) Imputation techniques to deal with attrition / missing values**		

For the intention-to-treat analysis, missing values in the data were replaced using multiple imputation by chained equations (with m=5 imputations). The pooled data (the mean of all 5 imputations) were calculated using the data imputed by linear regression. Subsequently, scale values were determined from the imputed and existing values. After data imputation, imputed and observed results were compared. The pooled imputed values proved to be more conservative, therefore, the results of imputed data set were used to evaluate the outcome of the web-based intervention.

12b) CONSORT: Methods for additional analyses, such as subgroup analyses and adjusted analyses

No specific subgroup analyses were performed

RESULTS

13a) CONSORT: For each group, the numbers of participants who were randomly assigned, received intended treatment, and were analysed for the primary outcome

Yes see Fig 1 in the manuscript

13b) CONSORT: For each group, losses and exclusions after randomisation, together with reasons

Yes it is displayed in Fig 1

13b-i) Attrition diagram

Yes, See Fig 1. in the manuscript

14a) CONSORT: Dates defining the periods of recruitment and follow-up

No, we did not report the exact time period

14a-i) Indicate if critical "secular events" fell into the study period

14b) CONSORT: Why the trial ended or was stopped (early)

The trial was not stopped earlier

15) CONSORT: A table showing baseline demographic and clinical characteristics for each group

Yes, See Tabl. 1 in the manuscript

15-i) Report demographics associated with digital divide issues

Yes, See Tabl. 1 in the manuscript

16a) CONSORT: For each group, number of participants (denominator) included in each analysis and whether the analysis was by original assigned groups

16-i) Report multiple "denominators" and provide definitions

Response, defined as the percentage of participants that had a reduction of depressive symptoms by 50% or more at postintervention (T3), was reached by 34.9% of all participants (n=140/401). In the guided group, the response rate was 48.3% (73/151), 43.3% (65/150) in the unguided group, and 2.0% (2/100) in the control group. Remission, defined as a postintervention BDI-II score of 12 or less, occurred in 25.4% of all participants (102/401) of the intention-to-treat sample. In the guided group, 39.7% of participants (60/151) reached remission, with 28.0% (42/150) in the unguided group. No participants in the control group reached remission.

16-ii) Primary analysis should be intent-to-treat

See results section with ITT sample		
**17a) CONSORT: For each primary and secondary outcome, results for each group, and the estimated effect size and its precision (such as 95% confidence interval)**		
Yes. See results section.		
**17a-i) Presentation of process outcomes such as metrics of use and intensity of use**		
A total of 301 participants received the intervention after baseline assessment. A mean of 9.4 (SD 2.3) modules were completed by each participant during the intervention period, and 254 participants (84.4%) completed the main course		
**17b) CONSORT: For binary outcomes, presentation of both absolute and relative effect sizes is recommended**		
For factor relationships, fewer participants (33/151, 22.0%) reported themselves to be married or living with a partner in the unguided group than in the control group (52/100, 52.0%; χ2<br>1=8.25, P=.01), whereas no difference was shown between the guided and control groups (χ2<br>1=1.56, P=.21) or between the guided and unguided groups (χ2<br>1=2.97, P=.08). More participants were employed in the guided group (82/151, 54.3%) and the unguided group (86/150, 57.3%) compared to those in the control group (57/100, 57.0%; guided vs control: χ2<br>1=9.12,<br>P=.01; unguided vs control: χ2<br>1=18.98, P<.001), while there was no difference between the guided and unguided groups (χ2<br>1=1.76, P=.18). More participants in the control group (25/100, 25.0%) were trainees than those in the guided group (12/151, 7.9%; χ2<br>1=5.68, P=.01) or unguided group (6/150, 4.0%; χ2<br>1=12.62, P<.001), while there was no difference between the guided and unguided groups (χ2<br>1=1.27, P=.26).<br>Lastly, more participants in the control group (14/100, 14.0%) than in the unguided group (3/150, 2.0%; χ2<br>1=6.55, P=.05)<br>reported other occupations.		
**18) CONSORT: Results of any other analyses performed, including subgroup analyses and adjusted analyses, distinguishing pre-specified from exploratory**		
Moderator analysis was used to analyze the influence of various sociodemographic variables on the primary outcome. Regression analysis was directed at explaining the changes in the BDI-II (the difference between T3 and T1 was used as a criterion).		
**18-i) Subgroup analysis of comparing only users**		

We reported both groups, completers and ITT		
**19) CONSORT: All important harms or unintended effects in each group**		
We could not observe any side effects by the intervention		
**19-i) Include privacy breaches, technical problems**		
**19-ii) Include qualitative feedback from participants or observations from staff/researchers**		
DISCUSSION		
**20) CONSORT: Trial limitations, addressing sources of potential bias, imprecision, multiplicity of analyses**		
**20-i) Typical limitations in ehealth trials**		
Additional treatment (12<br>people were in therapy and 70 were receiving psychiatric<br>treatment in both intervention groups) could have contributed<br>to the effects and possibly caused a reduction in internal validity.<br>Third, although conversations between psychotherapists and<br>participants were standardized in the guided group, we had no<br>insights into the actual conversations and whether the structure<br>of the predetermined content was followed.		
**21) CONSORT: Generalisability (external validity, applicability) of the trial findings**		
**21-i) Generalizability to other populations**		
First, using wide inclusion criteria, we acquired a<br>heterogeneous study sample [37]. Second, the option to receive<br>additional treatment impeded the attribution of treatment effects<br>solely on the web-based intervention. Additional treatment (12<br>people were in therapy and 70 were receiving psychiatric<br>treatment in both intervention groups) could have contributed<br>to the effects and possibly caused a reduction in internal validity		
**21-ii) Discuss if there were elements in the RCT that would be different in a routine application setting**		
**22) CONSORT: Interpretation consistent with results, balancing benefits and harms, and considering other relevant evidence**		
**22-i) Restate study questions and summarize the answers suggested by the data, starting with primary outcomes and process outcomes (use)**		
We investigated the efficacy of a guided and unguided<br>web-based intervention for the treatment of depressive disorders<br>and found a significant improvement of depressive symptoms<br>in the BDI-II (primary outcome) and the HRSD-24 for both<br>intervention groups compared with those in the control group<br>in the intention-to-treat sample, with large pre- and<br>postintervention difference effect sizes observed for each<br>intervention (BDI-II: guided group, d=1.44; unguided group,<br>d=1.38; HRSD-24: guided group, d=1.76; unguided group		
**22-ii) Highlight unanswered new questions, suggest future research**		
Other information		
**23) CONSORT: Registration number and name of trial registry**		

RR2-10.1186/s13063-021-05218-4		
**24) CONSORT: Where the full trial protocol can be accessed, if available**		
Krämer R, Köhler S. Evaluation of the online-based self-help programme "Selfapy" in patients with unipolar depression:<br>study protocol for a randomized, blinded parallel group dismantling study. Trials 2021 Apr 09;22(1):264 [FREE Full text]<br>[doi: 10.1186/s13063-021-05218-4] [Medline: 33836810		
**25) CONSORT: Sources of funding and other support (such as supply of drugs), role of funders**		
Yes: The study was funded by a commercial organization: Selfapy GmbH. RK worked for Selfapy as a student (November 2016 to<br>September 2017). SK, LKV, and AS have no relationship with Selfapy GmbH		
**X26-i) Comment on ethics committee approval**		
The study was approved by the ethics committee of the medical<br>faculty of the Charité University Medicine Berlin.		
**x26-ii) Outline informed consent procedures**		
**X26-iii) Safety and security procedures**		
acute suicidality (assessed<br>using HRSD-24; individuals were excluded if they had a score<br>≥3 on suicidality items). Individuals who were excluded from<br>the study due to illness severity were advised to seek<br>professional help. Additional details have been previously<br>published [13].		
**X27-i) State the relation of the study team towards the system being evaluated**		
